# Supplementary figures and images for: A denaturation-free protocol for in situ visualization of short nuclear DNA sequences using padlock probes with rolling-circle amplification
Source: PLoS One. 2025 Oct 28;20(10):e0335619. doi: 10.1371/journal.pone.0335619 (PMC12561931; doi:10.1371/journal.pone.0335619)

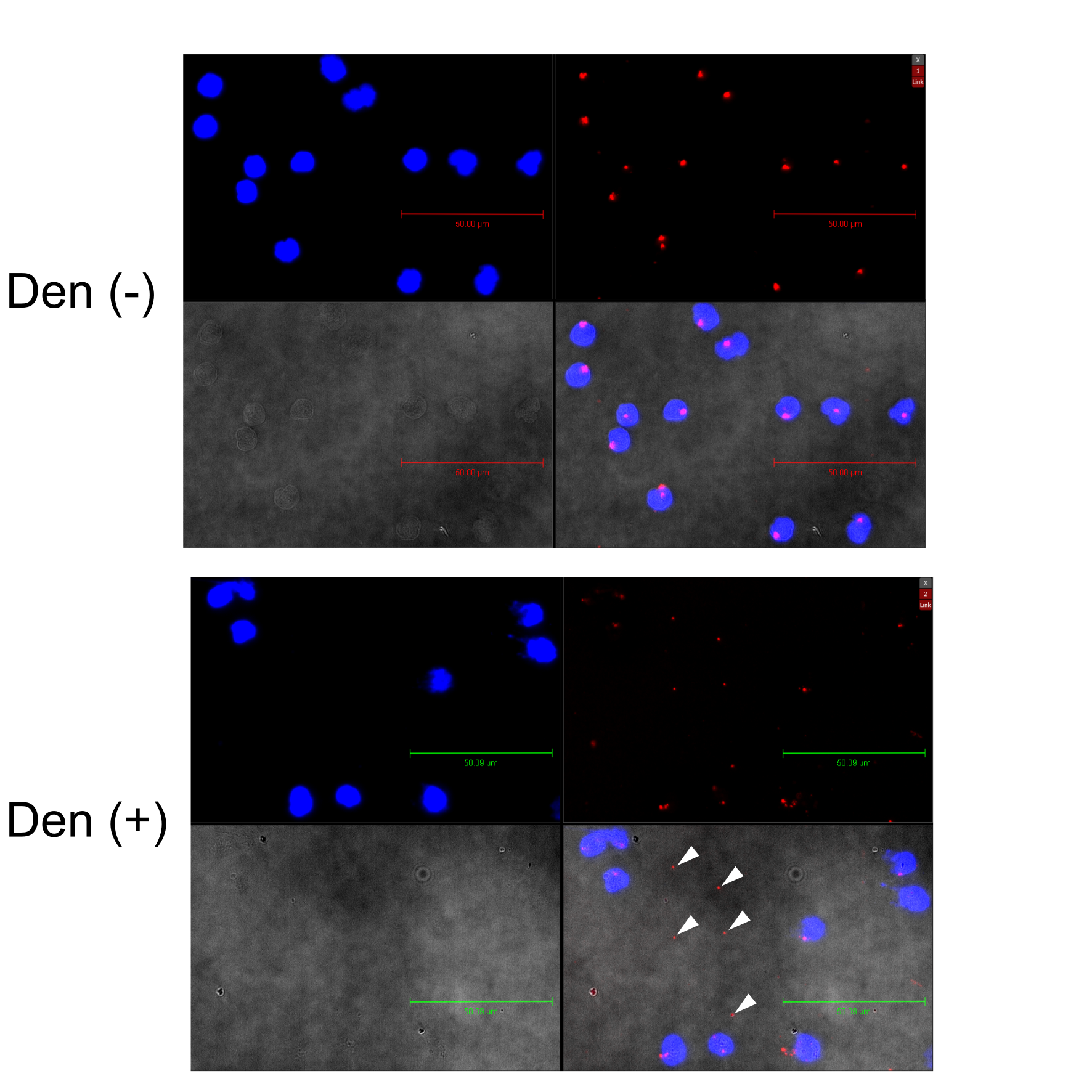

Supplement: S1 Fig — Detection of DYZ1 in male PBMCs without or with prior standard denaturation (Den – or Den + , respectively). The composite images consists of four panels: nuclear DNA staining (top left), RCA signal (top right), bright-field microscopy (bottom left), and the merged image (bottom right).White arrow heads indicate RCA signals locating clearly outside nuclei. (TIF) [file pone.0335619.s001.tif]

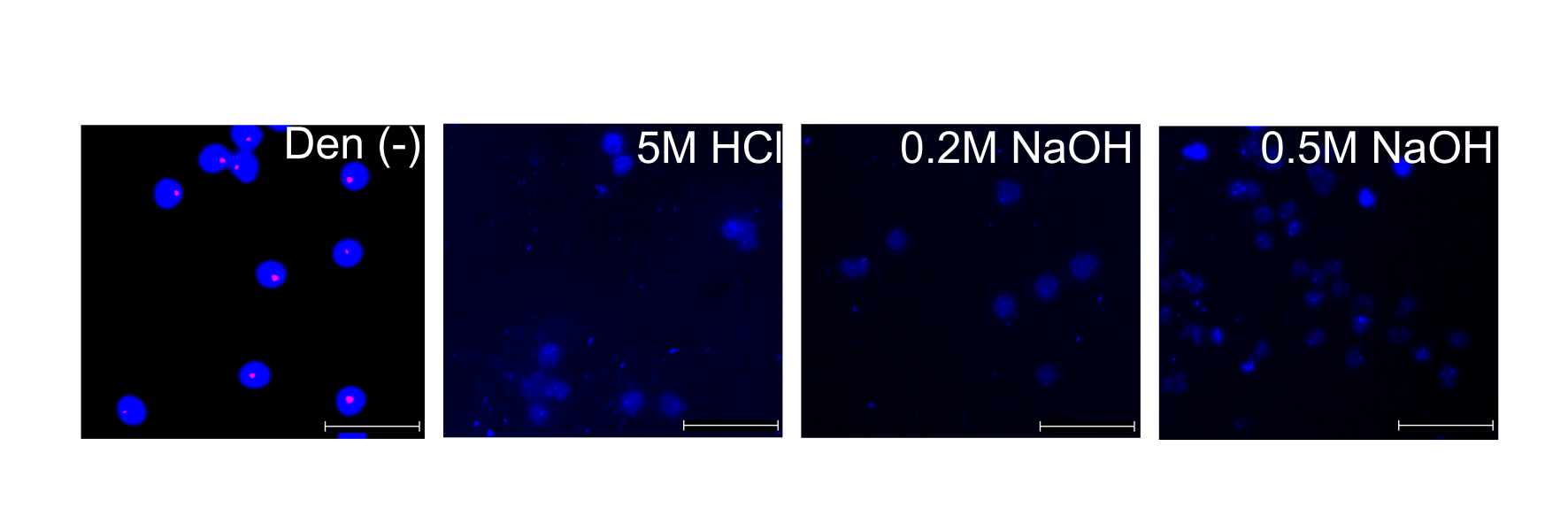

Supplement: S2 Fig — Detection of DYZ1 in male PBMCs without (Den –) or with prior denaturation by 5 M HCl, 0.2 M NaOH or 0.5 M NaOH, respectively. Scale bars represent 20 μm respectively. (TIF) [file pone.0335619.s002.tif]

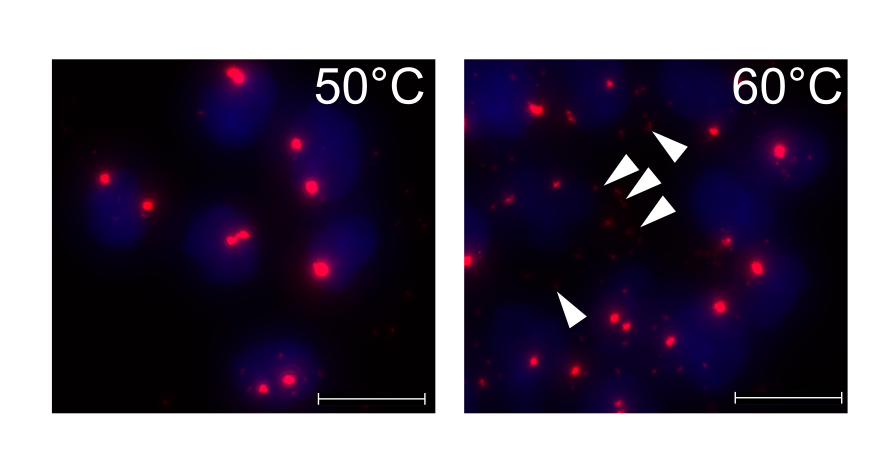

Supplement: S3 Fig — Detection of DYZ1 in Jurkat cells with combined padlock probe hybridization and ligation at the indicated temperatures, followed by RCA. White arrow heads indicate smaller RCA signals than the main signals. Scale bars represent 20 μm respectively. (TIF) [file pone.0335619.s003.tif]

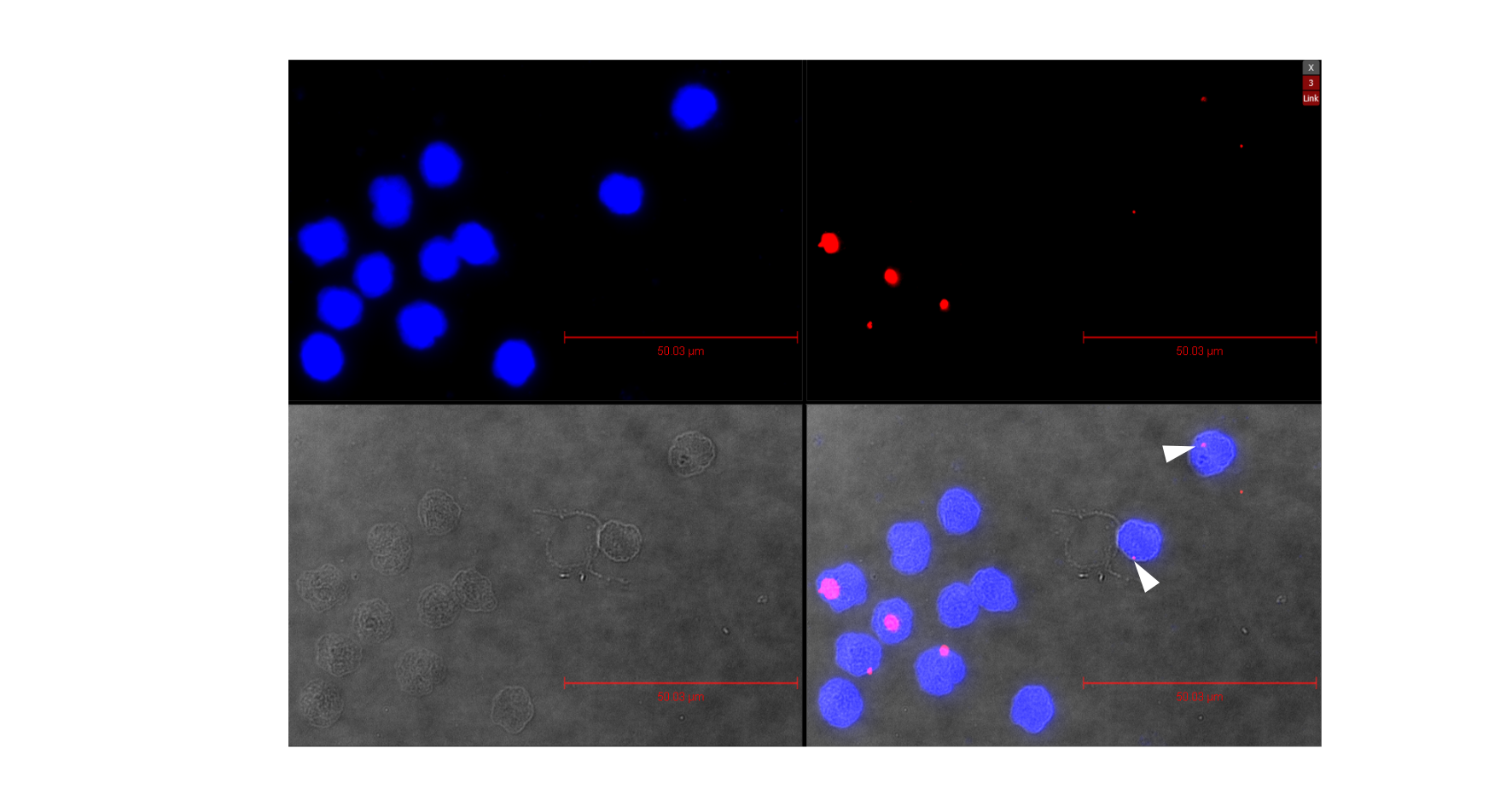

Supplement: S4 Fig — Detection of TMSB4Y in male PBMCs (M1). The composite images consists of four panels: nuclear DNA staining (top left), RCA signal (top right), bright-field microscopy (bottom left), and the merged image (bottom right). White arrow heads indicate smaller RCA signals than the main signals. (TIF) [file pone.0335619.s004.tif]
