# Supplementary material for: A denaturation-free protocol for in situ visualization of short nuclear DNA sequences using padlock probes with rolling-circle amplification
Source: PLoS One. 2025 Oct 28;20(10):e0335619. doi: 10.1371/journal.pone.0335619 (PMC12561931; doi:10.1371/journal.pone.0335619)
Supplement: Attachment — Submitted filename: PLOS ONE_Review.docx [file pone.0335619.s006.docx]

In this manuscript, the authors describe a denaturation-free method for in situ genomic DNA detection based on padlock probes and rolling-circle amplification (RCA). Short-lived openings in the DNA duplex are captured by circularizing oligonucleotides—padlock probes—that are enzymatically sealed by a DNA ligase in a sequence-specific manner. Once circularized and coiled around their target strands, the probes are amplified via RCA, yielding localized fluorescent signals for high-resolution visualization. Using this approach, genomic regions with thousands of repeats were detected in human leukocytes within a few hours, achieving over 99% efficiency and below 0.15% false positives. I think this work would meet the criteria of PLOS ONE. if the following issues are properly addressed.

1.Regarding the organization of the manuscript, Figure 1 presents actual sample detection results, while Figure 2 shows methodological validation. It is recommended to place the content of Figure 1 after Figure 4.

2.In Figure 2, the validation of DYZ1 specificity is not sufficiently rigorous. It is recommended to supplement with a blocking experiment, i.e., pre-blocking by adding DNA sequences complementary to the target sequence before performing dfPP.

3.The phrase "data not shown" appears multiple times in the text (e.g., line 179, line 184, line 227). It is suggested to include these data in the supporting information.

4.In Figure 2F, the signal dots are difficult to discern. Please provide a clear, enlarged image with the locations of the signal dots circled.

5.In Table 1, the detection efficiency for the minimum target length is reported as 13%-65%. How is the detection efficiency defined? Please add the calculation method to the experimental procedures section. Could the large variation in detection efficiency (13%–65%) be due to the small sample size (only 4 males and 1 female)? Since the detection efficiency for DYZ1 and Pal3 can exceed 95%, does this indicate that dfPP is only suitable for detecting repetitive regions and not applicable for specific short target sequences?
