# Supplementary material for: A denaturation-free protocol for in situ visualization of short nuclear DNA sequences using padlock probes with rolling-circle amplification
Source: PLoS One. 2025 Oct 28;20(10):e0335619. doi: 10.1371/journal.pone.0335619 (PMC12561931; doi:10.1371/journal.pone.0335619)
Supplement: Attachment — Submitted filename: 250929Response_To_Reviewers.docx [file pone.0335619.s007.docx]

PLOS One

Dear Reviewers:

We are grateful for the insightful and constructive feedback for our manuscript. We have addressed all the points raised and revised the manuscript as detailed below.

We look forward to hearing from you at your earliest convenience.

Sincerely,

Ryoyo Ikebuchi and Ulf Landegren

Molecular Tools and Functional Genomics, Department of Immunology, Genetics and Pathology, Uppsala University

ryoyo.ikebuchi@igp.uu.se	ulf.landegren@igp.uu.se

Uppsala Biomedical Center C10:2 Husargatan 3, Uppsala, Sweden, Box 815, SE-751 08

In this manuscript, the authors describe a denaturation-free method for in situ genomic DNA detection based on padlock probes and rolling-circle amplification (RCA). Short-lived openings in the DNA duplex are captured by circularizing oligonucleotides—padlock probes—that are enzymatically sealed by a DNA ligase in a sequence-specific manner. Once circularized and coiled around their target strands, the probes are amplified via RCA, yielding localized fluorescent signals for high-resolution visualization. Using this approach, genomic regions with thousands of repeats were detected in human leukocytes within a few hours, achieving over 99% efficiency and below 0.15% false positives. I think this work would meet the criteria of PLOS ONE. if the following issues are properly addressed.

1.Regarding the organization of the manuscript, Figure 1 presents actual sample detection results, while Figure 2 shows methodological validation. It is recommended to place the content of Figure 1 after Figure 4.

We have revised the manuscript in line with the reviewer’s comment. Figures 2, 3 and 4 in the original draft have been renumbered as Figure1, 2 and 3 in the revised version, while the original Figure 1 is now Figure 4. Additionally the data regarding dfPP specificity (originally presented as Figure 1H) has been relocated to Figure 3E to improve readability.

2.In Figure 2, the validation of DYZ1 specificity is not sufficiently rigorous. It is recommended to supplement with a blocking experiment, i.e., pre-blocking by adding DNA sequences complementary to the target sequence before performing dfPP.

Unfortunately, blocking experiments are not applicable to our assay, unlike RNA FISH, because the blocking oligonucleotide cannot access the double stranded target region (as shown in experiments where padlock probes were added in the absence of a ligase (new Fig. 1F left). Therefore, we have revised the sentence from “were indeed DYZ1-specific signals” to “were indeed chrY-specific signals” (Lines 128-129).

During this project, we considered performing FISH assay alongside dfPP to demonstrate padlock probe specificity by showing co-localization of FISH and dfPP signals. But the two assays cannot be combined because dfPP cannot be performed on cells that have undergone the denaturation steps required for FISH assays, as shown in Figure 1B, C and supporting figure 1A. This limitation motivated us to measure the spatial distances between signals from p22.31 and DXZ1 (Fig. 3E) to support the dfPP specificity.

3.The phrase "data not shown" appears multiple times in the text (e.g., line 179, line 184, line 227). It is suggested to include these data in the supporting information.

As suggested, we have included the imaging data related to denaturation conditions and ligation temperatures as supporting figure 1. Regarding FISH data, instead of presenting our own results, we have cited Reference 25 (Wilson et al., 2025), which provides high-quality karyotype data demonstrating within-culture mosaicism, including variations in chrY.

4.In Figure 2F, the signal dots are difficult to discern. Please provide a clear, enlarged image with the locations of the signal dots circled.

Following the reviewer’s suggestion, we have inserted white circles in enlarged images in Figure1G (Figure 2F in the original version) to improve clarity.

5.In Table 1, the detection efficiency for the minimum target length is reported as 13%-65%. How is the detection efficiency defined? Please add the calculation method to the experimental procedures section.

We have added an explanation for how detection efficiency is defined in lines 485-487, “The numbers of RCA products was then divided by the number of nuclei to calculate rate of false and correct positive signals in negative and positive samples, respectively”.

Additionally, we have included the average detection efficiency in line 230 and in Table 1, instead of presenting only the range of detection efficiencies.

Could the large variation in detection efficiency (13%–65%) be due to the small sample size (only 4 males and 1 female)?

We have added one more data point from a male sample, labeled as M5, in the revised Fig.3. The total sample size for TMSB4Y detection is now 5 males and 4 females (line 230-231). The M5 sample showed a detection efficiency of 32.4%, but a considerable variation remains with this larger number of investigated subjects. Additionally, we observed variability in the intensity of individual RCA signals from single copy targets in any samples. These findings suggest that unknown factors may still be influencing RCA kinetics in cell samples, as discussed in the third paragraph in Discussion.

Since the detection efficiency for DYZ1 and Pal3 can exceed 95%, does this indicate that dfPP is only suitable for detecting repetitive regions and not applicable for specific short target sequences?

As suggested, there are still limitations in detecting single copy targets using dfPP, likely due to lower detection efficiency against dsDNA-targets compared to ssDNA (Fig1G). Additionally, RCA may be compromised in tightly condensed chromatin (Fig. 3 and other publications [1,2]). However, these limitations can be overcome in certain contexts. As the reviewers suggests, and illustrated in the paper, detection of repetitive regions provides more opportunities for detection. As described in the Discussion (from line 378), application of dfPP for tracing clonal evolution in somatic tissues will involve monitoring multiple mutant cells compensating for the limited efficiency, and the technology also allows simultaneous targeting of several mutations to enhance detection efficiency for malignant cells. In Table. 1 we compare detection efficiency of dfPP to that of some of other DNA detection technologies. We note that dfPP has the advantage over other technologies that any sequence may be targeted as there are no requirement for specific sequence features and that mild dfPP procedure presented herein may prove advantages for simultaneous analyses of other, sensitive molecules in the samples (Line 328).

1.	Yaroslavsky AI, Smolina I V. Fluorescence Imaging of Single-Copy DNA Sequences within the Human Genome Using PNA-Directed Padlock Probe Assembly. Chem Biol. 2013;20: 445–453. doi:10.1016/j.chembiol.2013.02.012

2.	Zhang K, Deng R, Teng X, Li Y, Sun Y, Ren X, et al. Direct Visualization of Single-Nucleotide Variation in mtDNA Using a CRISPR/Cas9-Mediated Proximity Ligation Assay. J Am Chem Soc. 2018;140: 11293–11301. doi:10.1021/jacs.8b05309
